# Turbot reovirus (SMReV) genome encoding a FAST protein with a non-AUG start site

**DOI:** 10.1186/1471-2164-12-323

**Published:** 2011-06-20

**Authors:** Fei Ke, Li-Bo He, Chao Pei, Qi-Ya Zhang

**Affiliations:** 1 State Key Laboratory of Freshwater Ecology and Biotechnology, Institute of Hydrobiology, Chinese Academy of Sciences, Wuhan 430072, China

## Abstract

**Background:**

A virus was isolated from diseased turbot *Scophthalmus maximus *in China. Biophysical and biochemical assays, electron microscopy, and genome electrophoresis revealed that the virus belonged to the genus *Aquareovirus*, and was named *Scophthalmus maximus *reovirus (SMReV). To the best of our knowledge, no complete sequence of an aquareovirus from marine fish has been determined. Therefore, the complete characterization and analysis of the genome of this novel aquareovirus will facilitate further understanding of the taxonomic distribution of aquareovirus species and the molecular mechanism of its pathogenesis.

**Results:**

The full-length genome sequences of SMReV were determined. It comprises eleven dsRNA segments covering 24,042 base pairs and has the largest S4 genome segment in the sequenced aquareoviruses. Sequence analysis showed that all of the segments contained six conserved nucleotides at the 5' end and five conserved nucleotides at the 3' end (5'-GUUUUA ---- UCAUC-3'). The encoded amino acid sequences share the highest sequence identities with the respective proteins of aquareoviruses in species group *Aquareovirus *A. Phylogenetic analysis based on the major outer capsid protein VP7 and RNA-dependent RNA polymerase were performed. Members in *Aquareovirus *were clustered in two groups, one from fresh water fish and the other from marine fish. Furthermore, a fusion associated small transmembrane (FAST) protein NS22, which is translated from a non-AUG start site, was identified in the S7 segment.

**Conclusions:**

This study has provided the complete genome sequence of a novel isolated aquareovirus from marine fish. Amino acids comparison and phylogenetic analysis suggested that SMReV was a new aquareovirus in the species group *Aquareovirus *A. Phylogenetic analysis among aquareoviruses revealed that VP7 could be used as a reference to divide the aquareovirus from hosts in fresh water or marine. In addition, a FAST protein with a non-AUG start site was identified, which partially contributed to the cytopathic effect caused by the virus infection. These results provide new insights into the virus-host and virus-environment interactions.

## Background

Aquareoviruses have been isolated from a wide variety of aquatic animals [1,2]. These viruses represent a great threat to the aquaculture industry in China and East Asia. As a genus of the family *Reoviridae*, viruses in *Aquareovirus *have eleven-segmented dsRNA genomes. The virus particles are icosahedral in symmetry and have a double-layered capsid. Aquareoviruses have been divided into seven species (*aquareovirus *A to G, AQRV-A to G) according to RNA-RNA blot hybridization or sequence comparison [3,4]. There are three aquareoviruses that have complete sequence information: Grass carp reovirus (GCRV, species AQRV-C), Golden shiner reovirus (GSRV, species AQRV-C), and American grass carp reovirus (AGCRV, species AQRV-G) [4-6]. In addition, nearly complete sequence data was available for Chum salmon reovirus (CHSRV, species AQRV-A) except for segment 4. Additionally, some other aquareoviruses have sequence information for parts of the genome segments. However, sequence and molecular data seems to be insufficient for comparing species in *Orthoreovirus*, which was considered as the most related genus with *Aquareovirus *[6].

The family *Reoviridae *contains fifteen genera of reoviruses with 9, 10, 11 or 12 dsRNA genome segments [4]. Members in *Orthoreovirus *(except MRV) and *Aquareovirus *make up the fusogenic reovirus, whose infection causes cell-cell fusion and the formation of a syncytium [7,8]. Up to now, the nonstructural fusion associated small transmembrane (FAST) proteins represent the only known nonstructural viral proteins that induce cell-cell fusion; however, they are not directly related to virus entry or exit. A number of FAST proteins have been identified in orthoreovirus and aquareovirus species, and the protein topology, structural motifs, and some key amino acids have also been identified [9-12]. Thus, the identification of new FAST proteins would help to the further elucidate their functions.

The only reported aquareovirus in China was grass carp reovirus (GCRV), which was isolated from fresh-water grass carp. Recently, a *Scophthalmus maximus *reovirus (SMReV) was isolated and identified from a diseased turbot. It is the first isolated aquareovirus from a marine fish in China. The complete genome sequence of SMReV was determined and compared with other reoviruses. Sequence and functional analysis also identified a FAST protein that utilized a non-AUG translation start site.

## Results

### Pathology, morphology, and the genome of SMReV

**Figure 1:**
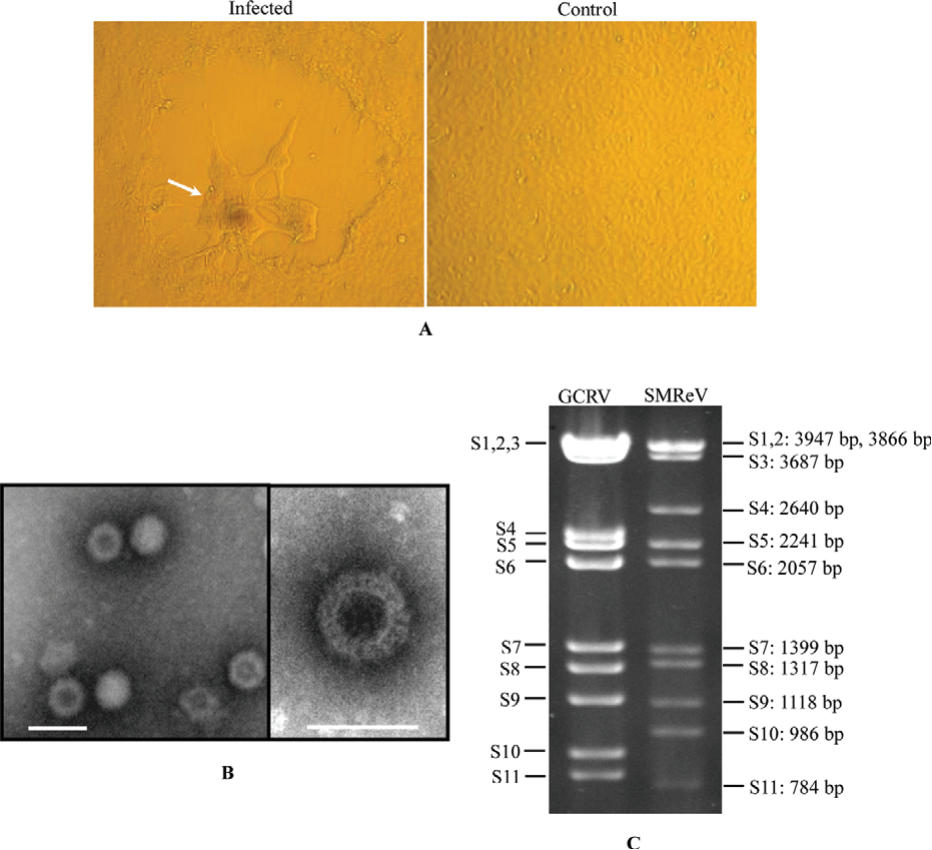
**Cytopathic effect, electron micrograph, and genome electrophoresis of SMReV**. (A) Cytopathic effect (CPE) induced by SMReV in GCF cell lines. Arrow indicated the syncytial. (×100). (B) Negatively stained virions. The bars represent 100 nm. (C) Purified genomic dsRNA of SMReV was analyzed by 1% agarose gel electrophoresis. Genomic dsRNA of GCRV-873 was used as control.

SMReV could cause a cytopathic effect (CPE) in Grass carp fins (GCF) and in Chinook salmon embryo (CHSE) cell lines after 4-5 days incubation. The optimal temperature was 20°C. The CPE contained several separate plaques in which syncytia formed (Figure 1A).

Electron microscopy observations showed that the negatively stained virions had the typical morphology of aquareoviruses, including an inner nucleocapsid surrounded by double-layered capsids, and were about 70-80 nm in diameter (Figure 1B). The biophysical and biochemical properties of SMReV included resistance to heat, acid (PH 3.0), and alkaline (PH11.0) treatment. Treatment with chloroform or 5-iodo-2'-deoxyuridine (IUdR, Sigma) did not affect the viral infectivity.

The SMReV genomes were purified and analyzed by 1% agarose gel electrophoresis. As shown in Figure 1C, the genome segments were separated into 10 distinct bands, with segments 1 and 2 comigrating. Comparison with the genome of GCRV-873 showed that migration of SMReV genome segments was different from those of GCRV.

The complete sequences of segments 1-11 of SMReV were obtained and have been deposited in GenBank under accession numbers HM989930-HM989940. The lengths of SMReV genome segments ranged from 784 (S11) to 3947 (S1) bp, with a total of 24042 bp (Table 1).

**Table 1:** Characteristics of genome segments and predicted functions of proteins in SMReV.

	Gene					Protein				Predicted function
			
Genome segment	Segment length (bp)	GC%	5'UTR (bp)	3'UTR (bp)	Nucleotide position of ORF	Coding potential	Protein size (aa)	MM (KDa)	Isoelectric point (pI)	
S1	3947	54.32	13	40	14-3907	VP1	1297	141.4	6.13	Core protein, capping enzyme
S2	3866	54.86	12	29	13-3837	VP2	1274	140.97	8.39	Core protein, polymerase
S3	3687	55.19	18	39	19-3648	VP3	1209	131.10	6.10	Core protein, Helicase, NTPase
S4	2640	57.54	24	162	25-2478	NS88	817	87.80	6.18	Nonstructural protein, involved in the formation of viral inclusion bodies with NS38
S5	2241	53.59	21	27	22-2214	VP4	730	80.52	7.23	NTPase
S6	2057	54.93	28	67	29-1990	VP5	653	69.25	4.69	Outer capsid
S7	1399	54.82	16	74	17-613	NS22	198	22.15	8.93	FAST protein
					489-1325	NS32	278	31.81	6.16	Nonstructural protein
S8	1317	55.50	12	51	13-1266	VP6	417	45.18	8.91	Core protein
S9	1118	56.26	25	40	26-1078	NS38	350	38.12	6.60	Nonstructural protein, involved in formation of viral inclusion bodies with NS88
S10	986	56.80	27	62	28-924	VP7	298	32.18	7.56	Outer capsid
S11	784	56.63	24	52	25-732	NS25	235	25.32	7.88	Nonstructural protein

### Non-coding regions of SMReV genome segments

As shown in Table 1, the lengths of SMReV non-coding regions ranged from 12 to 28 nucleotides at the 5' end and ranged from 27 to 162 nucleotides at the 3' end. The S4 genome segment had a non-coding region as long as 162 nucleotides at the 3' end, which was longer than the corresponding segment any other aquareoviruses so far sequenced.

Conserved terminal nucleotide sequences have been considered as a feature in reovirus classification. Comparison of the genome sequences of SMReV showed that all of the segments had conserved terminal sequences. The conserved nucleotides 5'-GUUUUA^U^/G/_A_-3' were present at the 5' ends in all the positive strands of each segment and 5'-^U^/_A_^U^/_A_UCAUC-3' was present at the 3' end. They were very similar to those of CHSRV (AQRV-A) (5'-GUUUUA^U^/_G_-3' at 5' end and 5'-^U^/_A_^U^/_A_UCAUC-3' at 3' end) and AGCRV (AQRV-G) (5'-GUUUUA^U^/_A_-3' at 5' end and ^U^/A/_C _^U^/_A_UCAUC-3' at 3' end) (Table 2). However, there were some differences between SMReV and species in AQRV-C (GCRV-873 and GSRV, 5'-GUUAUU^U^/_G_-3' at 5' end and 5'-^U^/_A_UCAUC-3' at 3' end). Interestingly, a newly isolated aquareovirus from grass carp showed a distinct terminal sequence at 5' end (GCRV HZ08, 5'-GUAAUU-3') [13]. Conserved terminal sequences could be used in genome assembly and packaging as "sorting" signals [14].

**Table 2:** Conserved terminal nucleotide sequences and percent sequence identities of genome segments and proteins between SMReV and other aquareovirus and orthoreovirus species.

		S1		S2		S3		S4		S5		S6		S7		S8		S9		S10		S11		Conserved terminal nucleotide sequences
	
		nt	aa	nt	aa	nt	aa	nt	aa	nt	aa	nt		nt	aa	nt	aa	nt	aa	nt	aa	nt	aa	
	
AQRV-A	CHSRV	74	84	79	93	79	85	-	-	72	80	75	85	74	61,50	79	88	78	85	71	76	83	-	5'-GUUUUA......UCAUC-3'
	TFRV	-	-	-	-	-	-	-	-	-	-	77	91	-	-	-	-	-	-	77	84	84	86	5'-GUUUUA......UCAUC-3'
	SBRV	-	-	-	-	-	-	-	-	-	-	-	-	-	-	78	86	-	-	77	84	-	-	5'-GUUUUA......UCAUC-3'
	ASRV-TS	-	-	79	94	-	-	-	-	-	-	-	-	-	-	-	-	-	-	73	78	-	-	5'-GUUUUA......UCAUC-3'
	ASRV-2009	-	-	-	-	-	-	-	-	-	-	-	-	78	73,76	-	-	-	-	77	84	-	-	5'-GUUUUA......UCAUC-3'
AQRV-C	GCRV-873	55	44	60	59	57	52	13	26	17	35	57	53	3	23,20	36	44	54	41	5	13	3	19	5'-GUUAUU......UCAUC-3'
	GSRV	56	44	60	59	58	52	13	26	17	35	60	53	3	25,21	38	45	54	41	5	13	5	20	5'-GUUAUU......UCAUC-3'
AQRV-G	AGCRV	52	44	60	58	57	53	13	23	10	35	56	51	5	21,26	38	40	25	37	8	17	7	23	5'-GUUUUA......UCAUC-3'
	GCRV-HZ08	6	30	37	42	9	34	-	-	2	22	4	29	-	-	-	-	-	-	-	-	-	-	5'-GUAAUU......UCAUC-3'
Orthoreovirus	MRV-1	5	25	28	40	7	31	2	12	2	17	3	25	2	NE,8	2	22	2	11	3	8	NE	NE	5'-GCUA...........UCAUC-3'
	MRV-2	3	24	24	40	3	31	1	13	2	18	4	25	2	NE,8	1	19	2	13	3	8	NE	NE	5'-GCUA...........UCAUC-3'
	MRV-3	2	25	28	40	12	31	1	12	2	17	3	25	2	NE,4	2	20	3	11	3	8	NE	NE	5'-GCUA...........UCAUC-3'
	ARV S1133	4	24	24	41	7	30	3	16	2	16	6	23	1	6,5	2	17	3	13	3	9	NE	NE	5'-GCUUUU.......UCAUC-3'

nt: nucleotide sequence

aa: amino acid sequence

"-": complete sequence not available

NE: no equivalent sequence

Abbreviations: CHSRV: Chum salmon reovirus; TFRV: Threadfin reovirus; SBRV: Striped bass reovirus; ASRV-TS: Atlantic salmon reovirus TS; ASRV-2009: Atlantic salmon reovirus 2009; GCRV-873: Grass carp reovirus 873; GSRV: Golden shiner reovirus; AGCRV: American grass carp reovirus; GCRV-HZ08: Grass carp reovirus HZ08; MRV-1: Mammalian orthoreovirus 1; MRV-2: Mammalian orthoreovirus 2; MRV-3: Mammalian orthoreovirus 3; ARV S1133: Avian orthoreovirus S1133

**Figure 2:**
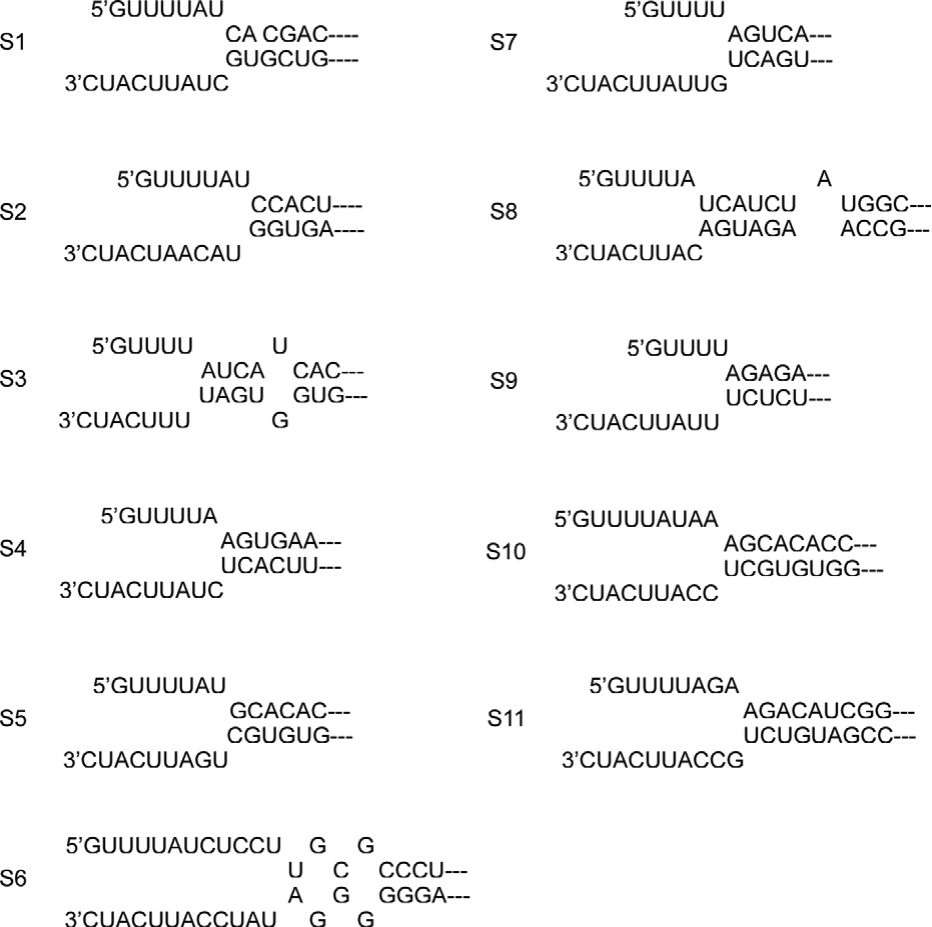
**Potentially imperfect inverted repeats at terminal nucleotide sequences of positive-sense RNA of SMReV genome segments**.

Moreover, the first and last nucleotides of each segment in all aquareoviruses were complementary (G-C). Potential imperfect inverted repeats were also predicted in the sequences adjacent to each termini of the SMReV positive-sense strand (Figure 2). It had been reported that complementary sequences in the 5' and 3' NCR could facilitate viral replication by circularizing the RNA transcript [15].

### Proteins encoded by genome segments S1-S6 and S8-S11

The S1 genome segment of SMReV was predicted to encode the core spike protein VP1, which functions as the mRNA capping enzyme. Four conserved amino acids, two lysines and two histidines, were found in the N-terminus of VP1 (lysine 176 and 196, histidine 229 and 238). The VP1 proteins of SMReV and GCRV shared a sequence identity of 44% (table 2) and were highly similar in their hydrophobic profiles.

The S2 genome segment of SMReV was predicted to encode the core protein VP2, which is an RNA-dependent RNA polymerase (RdRp). The catalytic domain of RdRp was identified between amino acids 550 and 798 in VP2 by motifscan (ExPASy proteomics server). Previous research had identified five important motifs, motif A, B, C, D and E, in RdRp [16]. Motif A (DXXXXD, 591-596), motif B (SG, 648-649), and motif C (GDD, 739-741) were found in the predicted catalytic domain of SMReV VP2. Amino acid alignments revealed that these motifs are also conserved in the RdRp proteins of *Aquareovirus *and *Orthoreovirus *species. Moreover, a hydrophobic region was identified in the C-terminus of SMReV VP2 that could be motif E of RdRp.

The S3 genome segment of SMReV was predicted to encode the core protein VP3, which functions as a helicase and NTPase. A zinc finger C2H2 domain was identified in the SMReV VP3 at amino acid positions 113-136, which is known to bind RNA. As revealed by Cryo-EM analysis, amino acids Glu502, Ser503, Thr504, and Thr505 are involved in RNA transcription in GCRV [17]. Amino acid alignments showed that the four amino acids were conserved in MRV, GCRV, GSRV, and AGCRV. However, the corresponding amino acids were Glu, Thr, Thr, and Thr in SMReV and CHSRV.

The S4 genome segment was predicted to encode the nonstructural protein NS88. This segment was larger than its homologs in other *Aquareovirus *species. The high percentage of G+C in the 5' portion and the complicated secondary structure of the genomic RNA made it difficult to determine the complete sequence of this segment. In this study, reverse transcriptase that was stable at 65°C was used in 5' RACE to clone the 5' part of S4 genome segment. It was anticipated that NS88 was necessary to form viral inclusion bodies during virus infection, in which the virus genome replication and virion morphogenesis occurs. There were two coils (amino acid residues positions 587-635 and 700-762) in NS88, as predicted by Coils program. Sequence analysis also revealed that the corresponding NS88 proteins in *Aquareovirus *species all contained the two coils regions, and conserved histidine and cysteine.

The S5 genome segment was predicted to encode the minor core protein VP4, which is thought to be a nucleoside triphosphate phosphohydrolase as a putative cofactor of VP2. Amino acid sequence alignments showed two conserved lysine residues (positions 409 and 413) in SMReV VP4, which were also conserved in MRV, ARV, and *Aquareovirus *species except CHSRV.

The S6 genome segment was predicted to encode the outer capsid protein VP5. An autolytic cleavage site was predicted to be located between amino acid residues Asn42 and Pro43. In addition, a myristoylation consensus sequence, which is essential for the autolytic cleavage, was located in the N-terminus of VP5. Sequence alignment revealed that the N-terminal sequence of VP5 in aquareovirus species was highly conserved.

The S8 genome segment of SMReV was predicted to encode the core protein VP6. VP6 has an amino acids sequence identity of about 20% with the σ2 protein of MRV species (Table 2). Secondary structure predictions revealed that a large number of β-sheets and turns existed in the N-terminal portion (75% of the protein) of SMReV VP6, which are characteristics of σ2/σA proteins in MRV and ARV species [18,19]. However, there were some differences in the C-terminal regions between SMReV VP6 and MRV σ2, as revealed by hydrophobic analysis. Most of the amino acid residues in the C-terminal regions of VP6 are hydrophobic; however, they are hydrophilic in σ2.

The S9 genome segment of SMReV was predicted to encode the nonstructural protein NS38. NS38 is thought to be involved in the formation of viral inclusion bodies with NS88. The amino acids sequence identity between NS38 and MRV σNS is lower than 20%, but they show a high similarity in secondary structure and in their hydrophobicity.

The S10 genome segment of SMReV was predicted to encode the major outer capsid protein VP7. As the major outer capsid protein, VP7 had the most variability among aquareovirus species groups.

The S11 genome segment of SMReV was predicted to encode the nonstructural protein NS25. No equivalent proteins of NS25 were found in reovirus species other than aquareoviruses by BLAST analysis. An immunofluorescense assay showed that NS25 is distributed in the cytoplasm during SMReV infection (data not shown).

### Genome segment S7 encodes a FAST protein

**Figure 3:**
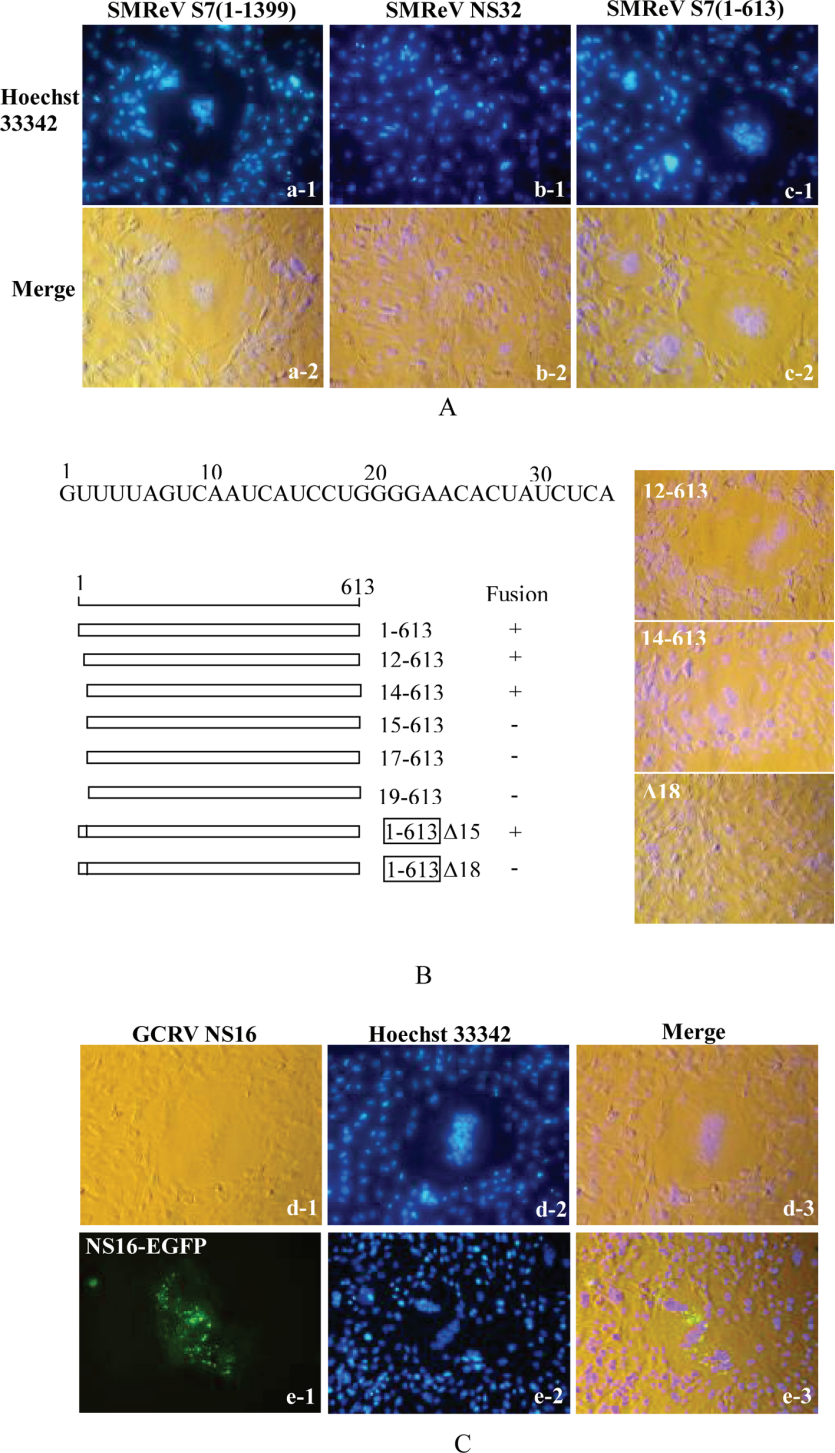
**FAST proteins encoded by SMReV and GCRV**. (A) Genome segment S7 of SMReV encodes a FAST protein. CIK cells were transfected with plasmids expressing the full length, 5' fragment (1-613), or NS32 of S7, respectively. Expression of full length or 5' fragment (1-613) of S7 induced syncycium formation (panel a 1-2 and panel c 1-2), but no syncycium formation was observed in cells expressing NS32 (panel b 1-2). (B) Determining the translation start site of NS22. The 5' terminal sequence of S7 is shown at top of the figure. The 5' fragment of S7 is schematically indicated by a horizontal line comprising bases 1-613 (positions numbered on the top). A similar horizontal bar indicates each truncation or mutation. The ability of each construct to form a syncytium is indicated as positive (+) or negative (-). Representative images of transfected CIK cells are presented at the right side of the figure. (C) Cell-cell fusion induced by NS16 encoded by S7 of GCRV. CIK cells were transfected with plasmid pcDNA3.1-NS16 and pEGFP-NS16 respectively.

Initial ORF analysis by EditSeq in DNASTAR software showed that SMReV genome segment S7 contained only one ORF, which started from an AUG codon at nucleotide 489. However, except for CHSRV, the S7 segment in aquareoviruses usually contains more than one ORF. To determine the ORFs contained by SMReV S7, different recombinant plasmids that contained all or part of the S7 segment cDNA were constructed and expressed in fish cell lines. When the entire S7 segment cDNA was expressed, cultured fish cells formed syncytia in which the nucleus aggregated (Figure 3A-a). However, the expression of cDNA (489-1325, NS32) did not cause cell-cell fusion (Figure 3A-b). Cells expressing cDNA (1-613) formed syncytia (Figure 3A-c). This indicated that the 5' part of the SMReV S7 segment encoded a protein that could cause cell-cell fusion and was translated from a non-AUG translation start site. Furthermore, expression of S7 cDNA (12-613) and cDNA (14-613) could induce cell-cell fusion (Figure 3B), but no cell-cell fusion occurred when cDNAs (15-613) and (19-613) were expressed. In addition, a point mutation at nucleotide 15 (^14^AUC^16 ^to ACC) did not influence the ability to form syncytia. However, syncytium formation was ablated by a point mutation at nucleotide 18 (^17^CUG^19 ^to CCG) (Figure 3B). Considering the consensus sequence (RNNAUGG) of the non-AUG start site [20] and the sequence (^14^AUCCUGG^20^) near the nucleotide position 17 of S7 segment, it can be concluded that SMReV S7 segment encoded a protein from a CUG translation start site. This ORF starts from a CUG codon at nucleotides 17-19 and is terminated by UGA at nucleotides 611-613.

Thus, the S7 genome segment of SMReV contained more than one ORF. The first ORF encoded the FAST protein NS22 and the second ORF was predicted to encode a nonstructural protein, NS32.

### Fusogenic proteins in SMReV and GCRV

**Figure 4:**
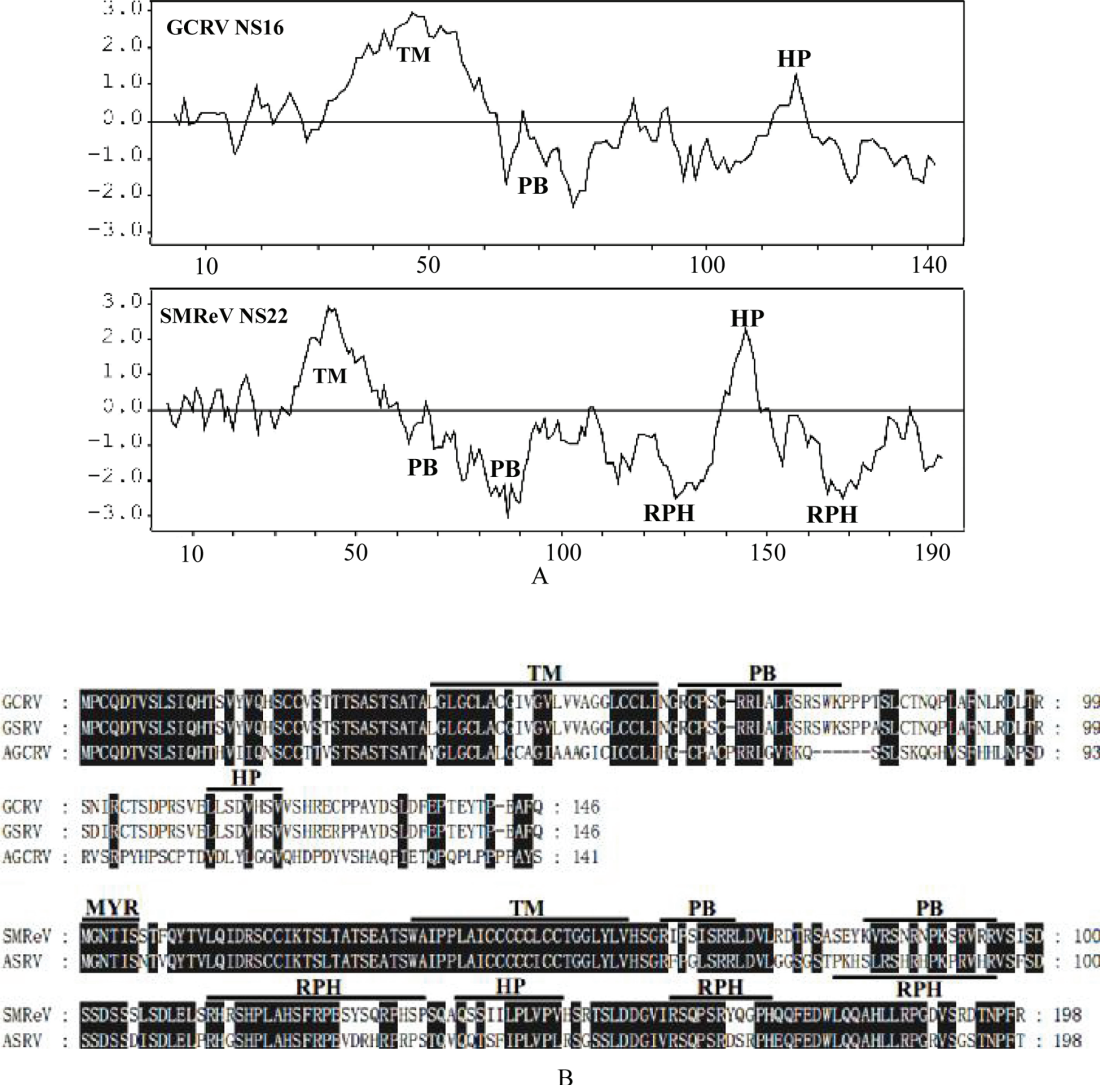
**Comparison of the structural features of FAST proteins in aquareoviruses**. (A) The hydrophobic characters of NS16 and NS22 were predicted by ProScale according to the algorithm of Kyte and Doolittle, with a window of nine residues. Amino acids residues with positive scores are hydrophobic and negative are hydrophilic. The numbers at the bottom indicate amino acid position. (B) Predicted structural motifs contained in FAST proteins of aquareoviruses. Highly conserved residues are marked by a black background. Motifs are marked with a black line. MYR, myristoylation consensus sequence; TM, transmembrane domain; HP, hydrophobic patch; PB, polybasic region; RPH, regions rich in arginine, proline and histidine.

In addition to NS22 of SMReV, the NS16 protein encoded by the first ORF (nucleotides positions 14-454) of the S7 genome segment of GCRV-873 was also identified here as a FAST protein. *In vitro *expression of NS16 in fish cells induced syncytium formation (Figure 3C-d). Expression of NS16-EGFP recombinant protein also induced syncytium formation, while NS16-EGFP was distributed in the fused cells (Figure 3C-e). Hydrophobic analysis by ProtScale (ExPASy Proteomics Server) revealed that NS22 and NS16 had a similar hydropathy profile (Figure 4A). However, there were some differences in the motifs contained in NS22 and NS16. A myristoylation consensus sequence (MGXXXS) was found in the N-terminus of NS22, but, surprisingly, no myristoylation site was predicted to exist in NS16. It had been reported that N-terminal myristoylation was necessary for the fusion activity of reptilian reovirus (RRV) p14 protein [11]. NS22 and NS16 were both predicted to contain a transmembrane domain (TM, positions 35-57 for NS22 and 37-60 for NS16) by TMpred [21]. Following the TM domain, there are regions that contain a stretch of basic amino acid residues (PB) in both proteins (positions 61-68 and 82-95 for NS22 and 63-78 for NS16). The polybasic regions are thought to support the translocation of the N-terminal domain (34 amino acids for NS22 and 36 amino acids for NS16) into face the extracellular environment [11]. Besides the TM domain, another hydrophobic region, the hydrophobic patch (PH), was predicted to exist in the C-terminal fragment of both proteins (positions 140-150 for NS22 and 113-119 for NS16). Moreover, there are two regions that are rich in arginine, proline, and histidine (RPH) in NS22 (Figure 4B). However, there are three RPH domains in the FAST protein of ASRV-2009 [12], despite its high sequence similarity with SMReV NS22.

### Comparison with other reovirus species and phylogenetic analysis

Comparison of the electropherotype of SMReV in agarose gel electrophoresis with those reported from other aquareoviruses [22] revealed that the electropherotype of SMReV was similar to the members in AQRV-A. Interestingly, it was different to the electropherotype of TRV, which was isolated from turbot in Spain [23].

The genome sequence and deduced amino acids were compared to the sequences available from other aquareoviruses. SMReV had a larger M4 genome (2640 bp) segment than other sequenced aquareoviruses (GCRV/GSRV, 2320 bp; AGCRV, 2293 bp). Overall identity values between SMReV and other aquareoviruses homologous proteins ranged from 13% to 94%. The highest identity was between SMReV and species in AQRV-A, for example, CHSRV, which ranged from 76.2% to 92.8%. However, the CHSRV genome sequence information lacked the complete M4 segment (Table 2).

**Figure 5:**
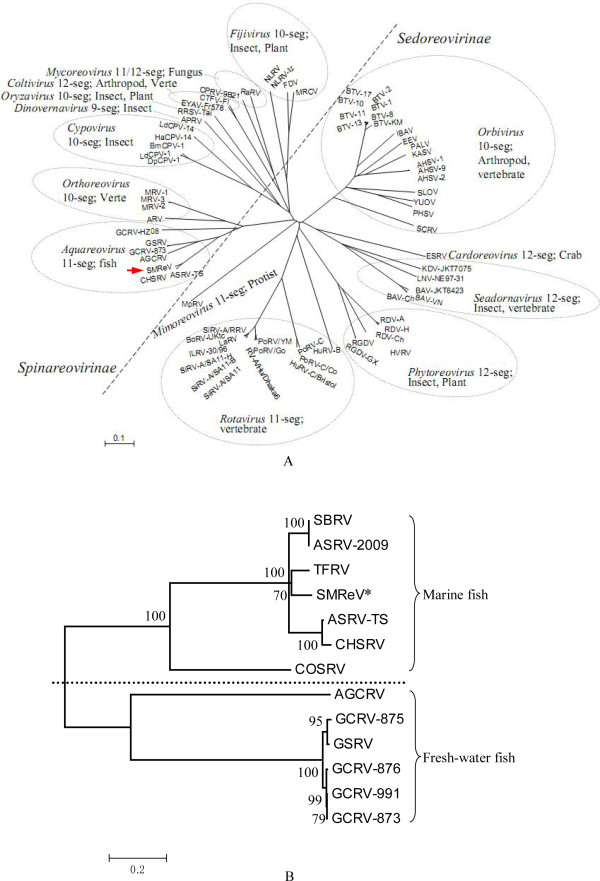
**Phylogenetic analysis**. (A) The phylogenetic analysis based on the RNA dependent RNA polymerase of *Reoviridae*. The phylogenetic tree was constructed using the neighbor-joining method in MEGA 4.0. Numbers of the genome segments and hosts of each genus are shown. Two subfamilies, *Spinareovirinae *and *Sedoreovirinae*, were divided by a skewed broken line. (B) The phylogenetic analysis of the major outer capsid protein VP7 from aquareovirus species was carried out as in materials and methods. The numbers given are frequencies (%) at which a given branch appeared in 1000 bootstrap replications. Viruses appearing above the broken line are from hosts that had seawater life-histories and viruses from hosts that lived in fresh water are listed below the broken line. GenBank accession numbers were collected in additional file 2 and 3.

The genes and proteins of SMReV were compared with their homologs from reovirus species other than aquareoviruses. The results showed that SMReV had a high similarity with species in *Orthoreovirus *(MRV and ARV, as shown in table 2). The highest amino acid sequence identities (40%) between SMReV and *Orthoreovirus *species (MRV and ARV) were observed in the RNA-dependent RNA polymerase (VP2 in SMReV, λ3 or λB in MRV and ARV species) (Table 2). A phylogenetic tree among members of fourteen genera of *Reoviridae*, for whom sequence information was available, was constructed based on the RdRp sequences (Figure 5A). The genus *Aquareovirus *was clustered more closely with the *Orthoreovirus*. Interestingly, as shown in Figure 5A, several genus groups that have different genome segments numbers and hosts have a relatively close evolutionary relationship. For example, members from the genus *Cardoreovirus *and *Seadornavirus *were closely clustered. Members from the genus *Cypovirus *and *Dinovernavirus *were also closely clustered. In addition, there were two subfamilies in *Reoviridae: Spinareovirinae *and *Sedoreovirinae*. Members in *Spinareovirinae *are turreted reoviruses, which have turrets situated on the virus core structure. Members in *Sedoreovirinae *are non-turreted reoviruses. Two large species groups also existed in the present phylogenetic analysis. On the left side of Figure 5A (divided by a skewed line), are members in *Spinareovirinae*. Members in *Sedoreovirinae *are clustered on the right side of Figure 5A.

As the viral major outer capsid protein, VP7 is the most variable protein in aquareoviruses. A phylogenetic tree was constructed with VP7 amino acid sequences in aquareoviruses. It showed that SMReV was most related to viruses in AQRV-A (SBRV, striped bass reovirus; ASRV; CHSRV; TFRV, threadfin reovirus) (Figure 5B). Interestingly, the viruses from hosts that had a seawater life history were closely related (above the broken line in Figure 5B), and were different from those whose hosts lived in fresh water (below the broken line in Figure 5B).

The phylogenetic information has been deposited in the TreeBASE database under access URL: http://purl.org/phylo/treebase/phylows/study/TB2:S11304.

## Discussion

### Putative functions of proteins revealed by motif comparison

It has been reported that the four conserved amino acids (two lysines and two histidines) in VP1 are essential amino acids for the guanylyltransferase activity of the homologous proteins in MRV, ARV (avian orthoreovirus), and GCRV [24-26]. Several functional domains were also identified in GCRV VP1 by CryoEM analysis, such as the GTPase domain, the methyltransferase domain, and the immunoglobulin domain [17].

The two lysine residues in VP4 are essential for ATPase activity in homologous protein μA of ARV [27]. By comparison with the proteins from MRV and ARV, VP4 was thought to be a nucleoside triphosphate phosphohydrolase and a putative cofactor of VP2.

The autolytic cleavage site, Asn42 and Pro43, which allowed the protein to be cleaved during virus infection to produce an N-terminal fragment and a C-terminal fragment [28], was identified as SMReV VP5. Previous research reported that VP5 and VP7 formed heterodimers to form the outer capsid in GCRV [29]. In the early stage of infection, the outer capsid is proteolytically cleaved and disassembled to form the infectious subviral particles (ISVPs), which have enhanced infectivity [30].

Viral inclusions, or viral factories, are formed in the cytoplasm during reovirus infection. It has been reported that MRV nonstructural proteins μNS and σNS form viral inclusion bodies *in vivo *and *in vitro *[31,32]. Based on sequence comparisons, it was anticipated that SMReV NS88 had a function in forming viral inclusions along with NS38. The two coils structure in NS88 was thought to be essential for viral inclusions formation. Conserved histidines and cysteines between the two coil regions are also important for inclusion formation in MRV and ARV [33,34]. In addition, the N-terminal amino acid residues in NS38 were predicted to form an alpha-helix, which could be important for nucleoprotein complex formation in the σNS protein [35].

Moreover, the second ORF of S7 segment in MRV encodes the σC protein, which is a structural protein and is involved in cell attachment [36]. However, the protein corresponding to σC in *Aquareovirus *species is a nonstructural protein [29].

### FAST proteins of aquareoviruses with different translation start sites

Sequence and structural analysis agreed with the previous report that there were two types of FAST proteins in aquareoviruses [12]. One type comprises the NS16 proteins encoded by GCRV, GSRV (AQRV-C), and AGCRV (AQRV-G). The other type comprises the NS22 proteins encoded by SMReV and ASRV-2009 (AQRV-A). The FAST proteins of SMReV and ASRV-2009 use a non-canonical translation start site. There are some differences in the motifs in the two types of FAST proteins (Figure 4B). Racine et al. reported that NS16 could be a homolog of the reptilian orthoreovirus (RRV) p14 FAST protein. However, p14 contains an N-terminal myristoylation site that is essential for fusion activity [11]. Sequence analysis found no N-terminal myristoylation site in NS16. This indicated that NS16 could not be a homolog of RRV p14. The sequence comparison also showed that the motif and structural arrangements of NS16 were more similar with ARV p10 FAST protein [9]. Further analysis of the functions of each motif could facilitate the understanding of the mechanisms involved in syncytium formation and the evolution of FAST proteins.

Non-AUG translation start codons have been reported in many organisms, including viruses [20,37], such as the Sendai virus [38] and the Moloney murine leukemia virus (MoMuLV) [39]. Recently, a CUG start codon was identified in ASRV-2009, which was utilized for translating a FAST protein [12]. The non-canonical start site (CUG in SMReV and ASRV-2009) used in FAST proteins could be a strategy by which the translation efficiency is regulated. Inefficient expression of FAST proteins could reduce the rate of cell-cell fusion and facilitate the production of viral progeny. In addition, the start codon of the FAST protein of GCRV (NS16) is AUG; however, the nucleotide sequence around this AUG site (ACCAUGC) did not accord with the Kozak consensus sequence (G/ANNAUGG). This also indicated that NS16 could be translated with relatively low efficiency.

### Taxonomic status of SMReV and evolution analysis

Aquareoviruses have been divided into seven *Aquareovirus *species groups (AQRV-A to AQRV-G) [3,4]. The division of the different *Aquareovirus *species groups was based on electropherotype, serological comparison, the ability to reassort during mixed infections, conserved terminal sequences, and RNA sequence analyses. Genome electrophoresis, and gene and protein comparison revealed that SMReV could be a member of species group AQRV-A. Interestingly, turbot reovirus (TRV), which was isolated from Spain, was classified in species group AQRV-E by RNA-RNA hybridization [23].

Phylogenetic analysis indicated that the evolution of VP7 was affected by selective pressure from the host organisms. Evolution of aquareoviruses was closely related to the environment in which the host organisms lived. The family *Reoviridae *contains a large number of members that infect vertebrates, invertebrates, plants, and fungi. Members in *Aquareovirus *and *Orthoreovirus *have a common evolutionary origin with those from *Mycoreovirus *and *Coltivirus*, *Cypovirus*, and *Dinovernavirus*. It is interesting that viruses from vertebrates and fungi have a common ancestor. These genera comprise viruses that have genome segments ranging from 9 to 12. It has been speculated that reoviruses diverged from a common ancestor may have gained or lost a genome segment that was required or not in different hosts during the course of evolution [40]. In this case, the S11 segment in *Aquareovirus *species that has no equivalent in *Orthoreovirus *may be involved in virus-host interactions. Moreover, the model of genetic "jump", which involves changes in the number of genome segments, has been reported between the rotaviruses and the seadornaviruses, and between the aquareoviruses and the coltiviruses [4,41]. This model involves a process in which a single segment undergoes duplication and deletion to generate two separate segments. In this case, the S7 segment of aquareoviruses corresponds to segments 9 and 12 of coltiviruses.

## Conclusions

In summary, the present study provided the complete genome sequence of a newly isolated turbot reovirus from China. It is the first complete sequence of an aquareovirus from marine fish. Amino acids comparison and phylogenetic analysis suggested that SMReV is a new aquareovirus in the species group *Aquareovirus *A. Phylogenetic relationships among aquareoviruses revealed that VP7 could be used as a reference to divide the aquareovirus from freshwater hosts from those from marine hosts. Based on the complete genome sequence, a FAST protein with a non-AUG start site was identified, which partially contributed to the cytopathic effect caused by viral infection. These results provide new insights into the virus-host or virus-environment interactions.

## Methods

### Original viral isolate preparation

Diseased cultured turbot *Scophthalmus maximus *were sampled from a fish farm in Shandong province of northern China. The original viral isolate was prepared from tissues (liver, kidney, and spleen) of these fish as described previously [42]. Briefly, tissues were cut into pieces and homogenized in phosphate-buffered saline (PBS) containing antibiotics (penicillin, 100 IU ml^-1^; streptomycin, 100 IU ml^-1^). Extracts were filtered through a 45 um filter membrane and stored at -80°C as the original viral isolate for cell infections.

### Cell culture and virus infection

Chinook salmon embryo (CHSE), Flounder embryo (FE), *Epithelioma papulosum cyprini *(EPC), and GCF cell lines, were used for viral isolation and sensitivity tests. Tissue lysates acquired above were inoculated into confluent monolayers of these cells in 199 medium supplemented with 10% fetal bovine serum at 15°C, 20°C or 25°C.

Grass carp reovirus 873 (GCRV-873) used in this study was maintained in our laboratory [43]. *Ctenopharyngodon idellus *kidney (CIK) cells were used for GCRV-873 propagation at 25°C.

### Biophysical and biochemical property detection

The optimal temperature for virus propagation was assayed by infection of monolayers of GCF cell cultures at 15°C, 20°C, or 25°C. Heat stability was measured by incubating the virus suspension at 56°C or 60°C for 30 min, or 60 min and then the titer was determined. Chloroform and 5-iodo-2'-deoxyuridine sensitivity was determined as described previously [44].

### Virus isolation and purification

Infected GCF cells were harvested at five days post infection and centrifuged at 8, 000 g for 30 min at 4°C. The supernatant was then ultracentrifuged at 32, 000 rpm (Beckman rotor SW41) for 90 min. The virus pellet was resuspended in 1.5 ml 0.1 M Tris-Cl (PH 8.6) and further purified by discontinuous sucrose (20%30%, 40%, 50%, and 60%, w/v) gradient centrifugation at 30, 000 rpm for 60 min. The virus particle band was collected and sucrose was removed by centrifugation at 32, 000 rpm for 90 min in 0.1 M Tris-Cl (PH 8.6). The resulting pellet was resuspended in 0.2 ml 0.1 M Tris-Cl (PH 8.6) and stored at -20°C until use.

### Electron microscopy

Purified virus particles were negatively stained with 2% (w/v) uranylacetate and then examined with transmission electron microscopy (JEM-1230).

### Virus dsRNA preparation and cloning

Virus dsRNA was extracted from purified virus particles using Trizol Reagent (Invitrogen) following the manufacturer's protocols. Synthesis of cDNA from SMReV dsRNA was carried out using the single-primer amplification technique [45,46]. Briefly, an oligodeoxyribonucleotide primer, TC1 (5' PO_4_-CCCGCCATCCTCACTTAGACT-NH_2 _3') was ligated to both of the 3' ends of the dsRNA segments using T4 RNA ligase (Takara). dsRNA was denatured at 94°C for 5 min in the presence of 15% DMSO before being cooled rapidly on ice. cDNA synthesis was then carried out in a cDNA reaction using M-MLV (Promega). RNA was then removed by adding NaOH and the cDNA was annealed at 65°C overnight. After purification through a Sephacryl S-400 spin column (Promega), the cDNA was amplified by PCR using the primer TC2 (5' AGTCTAAGTGAGGATGGCGGG 3') with following cycles: 2 min elongation at 72°C; 94°C for 5 min; followed by 32 cycles of amplification (94°C for 30 s, 55°C for 30 s, 72°C for 3 min).

To clone the 5' parts of the S4 segment, cDNA was synthesized using a primer designed from the partial sequence obtained above, as described previously [47]. Reverse transcription was carried out using ThermoScript reverse transcriptase (Invitrogen). The resulting cDNA was purified and poly (C) tailed by terminal deoxynucleotidyl transferase (TDT, Takara). First round PCR was performed using primers designed above and 5' AP. The second round PCR was carried out using internal primers and 5' UP (see additional file 1).

### Sequencing and sequence analysis

PCR products were separated on a 1% agarose gel and all visible bands were purified and cloned into the pMD18-T vector (Takara). The positive clones were sequenced.

Aquareoviruses and other reoviruses sequences were obtained from the GenBank (NCBI). The accession numbers were collected in additional file 2. Nucleotide sequences and deduced amino acid sequences were analyzed using the EditSeq program (DNASTAR, USA). Multiple sequence alignments were conducted using the Clustal × 1.83 program. Sequence identities were calculated using the Clusta W method in the MegAlign program. Neighbor-joining phylogenetic trees were constructed using the Poisson correction models with 1000 bootstrap replicates in MEGA 4.0 [48]. Hydrophobicity plots of proteins were predicted using ProtScale (ExPASy) with the Kyte and Doolittle algorithm [49]. The coils program [50] was employed to predict coiled regions in SMReV protein.

### Plasmid construction

Genome segment S7 of SMReV was amplified from cDNAs obtained above using primers S7-F/R. The PCR products were digested with EcoR I and Xho I, and then ligated into vector pcDNA3.1(+) that had been digested with the same enzymes, which resulted in the recombinant plasmid pcDNA3.1-S7. To generate recombinant plasmids that contained truncations or mutations in the 5' portions of SMReV S7 segment, PCR primers that used pcDNA3.1-S7 as a template were designed and appear in Additional file 1. PCR products were cut and ligated into pcDNA3.1(+) vector with corresponding enzymes. NS32 of SMReV was also cloned into pcDNA3.1(+) using the same methods and primers NS32-F/S7-R.

To generate recombinant plasmid pcDNA3.1-NS16 and pEGFP-NS16, GCRV genomic dsRNA was used as template for RT-PCR. dsRNA of GCRV was reverse transcribed using the method described above. cDNA products were used in a PCR reaction to amplify NS16 using primers NS16-F/R or N3-NS16-F/R (Additional file 1), respectively. PCR products were cut and ligated into pcDNA3.1(+) or pEGFP-N3 with corresponding restriction enzymes.

All constructs were confirmed by DNA sequencing.

### Transfection and cell staining

CIK cells were seeded into 24-well or 6-well cell culture plates using 199 medium containing 5% of FBS for 24 h before transfection. Cells were transfected with plasmids using Lipofectamine 2000 (Invitrogen, U.S.A.) following the manufacturer's protocol.

Transfected cells were incubated 24 h at 25°C and then fixed and stained with Hoechst 33342 as described previously [51]. All samples were examined under a Leica DM IRB fluorescence microscope.

### Nucleotide sequence accession number

The GenBank/EMBL/DDBJ accession numbers for the sequences reported here are: HM989930-HM989940.

## Authors' contributions

ZQY designed the project; KF performed the experiments and analyzed the data; HLB and PC participated in PCR amplification and gene cloning; and KF and ZQY wrote the article. All authors read and approved the final manuscript.

## Supplementary Material

Additional file 1 Primer sequences used in 5' RACE and plasmids construction Click here for file

Additional file 2 **GenBank accession numbers of the aquareovirus and orthoreovirus genome sequences from this study**. Click here for file

Additional file 3 **GenBank accession numbers of the RNA dependent RNA polymerase in the family *Reoviridae***. Click here for file
