# Supplementary material for: Turbot reovirus (SMReV) genome encoding a FAST protein with a non-AUG start site
Source: BMC Genomics. 2011 Jun 20;12:323. doi: 10.1186/1471-2164-12-323 (PMC3135578; doi:10.1186/1471-2164-12-323)
Supplement: Additional file 1 — Primer sequences used in 5' RACE and plasmids construction [file 1471-2164-12-323-S1.DOC]

Additional file 1

Primer sequences used in 5’ RACE and plasmids construction (enzyme cleavage site was underlined, names contain del are deletion primers and contain mut are mutation primers. The mutation site are emphasized with italic type and putative translation start sites are marked with box)

| Primer | Primer sequences (5’-3’) | Primer | Primer sequences (5’-3’) |
| --- | --- | --- | --- |
| 5’AP | GGCCACGCGTCGACTAGTACGGGGGGGGGG | NS16-F | AATGAATTCAAACCATGCCTTGC (EcoR I) |
| 5’UP | GGCCACGCGTCGACTAGTAC | NS16-R | CGTCACTCGAGTCTGGTGTACTCAG (Xho I) |
| S7-F | AACGAATTCGTCAATCATCCTG (EcoR I) | N3-NS16-F | AATGAATTCAAACCATGCCTTGC(EcoR I) |
| S7-R | ATTCTCGAGTAAAGTCAACGGAAG (Xho I) | N3-NS16-R | TCAGGATCCCAACTGAAACGCCT(BamH I) |
| S7-1-F | ACTGGTACCGTTTTAGTCAATCATCCTG (Kpn I) | NS88-P1 | TAAGTGAACTGCTCGAGAAGATGGC (Xho I) |
| S7-1-R | AGTCTCGAGGATGAATAACAGTCACGACTG (Xho I) | NS88-P2 | AAGGGATCCCATGAGAAGGTCTGAC (BamH I) |
| S7-1-mut15-F | AAAGAATTCGTTTTAGTCAATCA***C***CCTGGGGAAC (EcoR I) | NS38-P1 | CACAAGCTTTAGACATGGCAAC (Hind III) |
| S7-1-mut18-F | AAAGAATTCGTTTTAGTCAATCATCC***C***GGGGAAC (EcoR I) | NS38-P2 | TGGGGTACCCTAGTTACCTCCC (Kpn I) |
| S7-1-del-11-F | TTTGGTACCTCATCCTGGGGAAC (Kpn I) | S7-1-del-14-F | AGTGGTACCTCCTGGGGAACACTAT (Kpn I) |
| S7-1-del-13-F | TTAGGTACCTATCCTGGGGAACAC (Kpn I) | S7-1-del-18-F | TCAATGGTACCGGGGAACACTATC (Kpn I) |
| NS32-F | AACGGTACCAGACGATGGAGTC (Kpn I) |  |  |
