# Supplementary material for: Turbot reovirus (SMReV) genome encoding a FAST protein with a non-AUG start site
Source: BMC Genomics. 2011 Jun 20;12:323. doi: 10.1186/1471-2164-12-323 (PMC3135578; doi:10.1186/1471-2164-12-323)
Supplement: Additional file 2 — GenBank accession numbers of the aquareovirus and orthoreovirus genome sequences from this study. [file 1471-2164-12-323-S2.DOC]

Additional file 2

GenBank accession numbers of the aquareovirus and orthoreovirus genome sequences used in this study.

| **Species** | **genome segment/accession number** | | |
| --- | --- | --- | --- |
| CHSRV |  |  | S7/ AF418300 |
|  | S1/AF418294; VP1/AAL31496 | S4/AF418297 | S8/ AF418301 |
|  | S2/AF418295; VP2/AAL31497 | S5/AF418298 | S9/ AF418302 |
|  | S3/AF418296; VP3/AAL31498 | S6/AF418299 | S10/ AF418303 |
|  |  |  | S11/ AF418304 |
| TFRV |  | S6/AY235428; VP5/AAP72182 | S10/ AY236219; VP7/AAP70008 |
|  |  |  | S11/ AF524892; NS25/AAP80767 |
| SBRV |  |  | S8/ AF450321; VP6/AAM93413 |
|  |  |  | S10/ AF450322; VP7/AAM93414 |
| ASRV-TS | S2/EF434978; VP2/ABO32573 |  | S10/ EF434979; VP7/ ABO32574 |
| ASRV-2009 |  |  | S7/FJ652575; p22/ACN38055; NS31/ACN38056 |
|  |  |  | S10/ FJ652576; VP7/ ACN38057 |
| COSRV |  |  | S10/U90430; VP7/AAC59609 |
| GCRV-873 |  |  | S7/AF403393; NS5/AAM92738; NS4/AAM92739 |
|  | S1/AF260511; VP1/AAG1043 | S4/AF403390; NS80/AAM92735 | S8/AF403394; VP6/AAM92740 |
|  | S2/AF260512; VP2/AAG10436 | S5/AF403391; VP4/AAM92736 | S9/AF403395; NS38-g/AAM92741 |
|  | S3/AF260513; VP3/AAG10437 | S6/ AF403392; VP5/AAM92737 | S10/AF403396; VP7/AAM92742 |
|  |  |  | S11/AF403397; NS3/AAM92743 |
| GCRV-875 |  |  | S10/ AF403409; VP7/AAM92756 |
| GCRV-876 |  |  | S10/AF403410; VP7/AAM92757 |
| GCRV-991 |  |  | S10/AF403411; VP7/AAM92758 |
| GSRV |  |  | S7/NC_005172; NS5/NP_938066; NS4/NP_938067 |
|  | S1/NC_005166; VP1/NP_938060 | S4/NC_005169; NS1/NP_938063 | S8/NC_005173; VP6/NP_938068 |
|  | S2/NC_005167; VP2/NP_938061 | S5/NC_005170; VP5/NP_938064 | S9/NC_005174; NS2/NP_938069 |
|  | S3/NC_005168; VP3/NP_938062 | S6/NC_005171; VP4/NP_938065 | S10/NC_005175; VP7/NP_938070 |
|  |  |  | S11/NC_005176; NS3/ NP_938071 |
| AGCRV |  |  | S7/NC_010590;NS16/YP_001837100; NS31/YP_001837101 |
|  | S1/NC_010584; VP1/YP_001837094 | S4/NC_010587; NS73/YP_001837097 | S8/NC_010591; VP6/YP_001837102 |
|  | S2/NC_010585; VP2/YP_001837095 | S5/NC_010588; VP5/YP_001837098 | S9/NC_010592; NS38/YP_001837103 |
|  | S3/NC_010586; VP3/YP_001837096 | S6/NC_010589; VP4/YP_001837099 | S10/NC_010593; VP7/YP_001837104 |
|  |  |  | S11/NC_010594; NS26/YP_001837104 |
| GCRV-HZ08 | S1/GQ896334 |  |  |
|  | S2/GQ896335 | S5/GQ896336 |  |
|  | S3/GU350742 | S6/GQ896337 |  |
| MRV-1 | S1/M24734; λ3/AAA47234 | S4/AF461682; μ2/ AAL99936 | S7/ M10260; σ1/AAA66877; σ1s/AAA66878 |
|  | S2/AF378003; λ2/AAK57507 | S5/AF490617; μ1/AAM10735 | S8/ M17598; σ2/ AAA47278 |
|  | S3/AF129820; λ1/AAD42304 | S6/NC_004257; μNS/NP_694610 | S9/ M18389; σNS/AAA47281 |
|  |  |  | S10/ M13139; σ3/AAA47272 |
| MRV-2 | S1/NC_004272; λ3/NP_694627 | S4/NC_004254; μ2/NP_694607 | S7/NC_004264;σ1/NP_694617; σ1s/NP_694618 |
|  | S2/NC_004260; λ2/NP_694613 | S5/NC_004270; μ1/NP_694625 | S8/NC_004263; σ2/NP_694616 |
|  | S3/ NC_004256; λ1/NP_694609 | S6/NC_004258; μNS/NP_694611 | S9/NC_004269; σNS/NP_694624 |
|  |  |  | S10/NC_004273; σ3/NP_694628 |
| MRV-3 | S1/NC_004282; λ3/NP_694687 | S4/NC_004280; μ2/NP_694685 | S7/NC_004277; σ1/NP_694682 |
|  | S2/NC_004275; λ2/NP_694680 | S5/NC_004278; μ1/NP_694683 | S8/NC_004279; σ2/NP_694684 |
|  | S3/NC_004274; λ1/NP_694679 | S6/NC_004281; μNS/NP_694686 | S9/NC_004283; σNS/NP_694688 |
|  |  |  | S10/NC_004276; σ3/NP_694681 |
| ARV S1133 | S1/AY641735; λA/AAV52819 | S4/AY639610; μA/AAV49511 | S7/AF330703; p10/AAK18186; σC/ AAK18188 |
|  | S2/DQ534201; λB/ABF82230 | S5/AY635934; μB/AAV34176 | S8/AF104311; σA/AAD17921  / |
|  | S3/DQ300175; λC/ABC01916 | S6/AY608700; μNS/AAT85608 | S9/ U20642; σB/AAA67065 |
|  |  |  | S10/U95952; σNS/AAB52598 |

Abbreviations: CHSRV: Chum salmon reovirus; AGCRV: American grass carp revirus; GCRV-873/875/876/991: Grass carp reovirus 873/875/876/991; GSRV: Golden shiner reovirus; GCRV-HZ08: Grass carp reovirus HZ08; TFRV: Threadfin reovirus; SBRV: Striped bass reovirus; ASRV-TS/2009: Atlantic salmon reovirus TS/2009; COSRV: Coho salmon reovirus; MRV-1/2/3: Mammalian orthoreovirus 1/2/3; ARV S1133: Avian orthoreovirus S1133.
