# Supplementary material for: Turbot reovirus (SMReV) genome encoding a FAST protein with a non-AUG start site
Source: BMC Genomics. 2011 Jun 20;12:323. doi: 10.1186/1471-2164-12-323 (PMC3135578; doi:10.1186/1471-2164-12-323)
Supplement: Additional file 3 — GenBank accession numbers of the RNA dependent RNA polymerase in the family Reoviridae. [file 1471-2164-12-323-S3.DOC]

Additional file 3

GenBank accession numbers of the RNA dependent RNA polymerase in the family *Reoviridae*.

| Genus | Virus  Full name (abbreviation) | Accession number |
| --- | --- | --- |
| *Orbivirus* | Bluetongue virus 13 (BTV-13) | AAA87363.1 |
|  | Bluetongue virus 11 (BTV-11) | AAA87362.1 |
|  | Bluetongue virus 10 (BTV-10) | CAA31306.1 |
|  | Bluetongue virus 17 (BTV-17) | AAA87364.1 |
|  | Bluetongue virus 2 (BTV-2) | AAA88823.1 |
|  | Bluetongue virus 8 (BTV-8) | CAM57242.2 |
|  | Bluetongue virus 1 (BTV-1) | ACR58458.1 |
|  | Bluetongue virus KM (BTV-KM) | AAS72882.1 |
|  | Ibaraki virus (IBAV) | BAD89093.1 |
|  | Equine encephalosis virus (EEV) | ACJ06234.1 |
|  | Palyam virus (PALV) | YP_052935.1 |
|  | Kasba (Chuzan) Virus (KASV) | BAA76549.1 |
|  | African horse sickness virus 1 (AHSV-1) | ACJ06244.1 |
|  | African horse sickness virus 9 (AHSV-9) | O70695.1 |
|  | African horse sickness virus 2 (AHSV-2) | ACI41990.1 |
|  | Stretch Lagoon orbivirus (SLOV) | YP_002925132.1 |
|  | Yunnan orbivirus (YUOV) | YP_443925.1 |
|  | Peruvian horse sickness virus (PHSV) | YP_460038.1 |
|  | St Croix River virus (SCRV) | YP_052942.1 |
| *Cardoreovirus* | Eriocheir sinensis reovirus (ESRV) | AAT11887.1 |
| *Seadornavirus* | Kadipiro virus JKT 7075 (KDV-JKT7075) | NP_694468.1 |
|  | Liao ning virus NE97-31 (LNV-NE97-31) | AAQ83562.1 |
|  | Banna virus JKT 6423 (BAV-JKT6423) | NP_694469.1 |
|  | Banna virus Vietnam (BAV-VN) | ACA50122.1 |
|  | Banna virus Ch (BAV-Ch) | AAF77631.1 |
| *Phytoreovirus* | Rice dwarf virus A (RDV-A) | BAA14222.1 |
|  | Rice dwarf virus H (RDV-H) | BAA01074.1 |
|  | Rice dwarf virus Chinese strain (RDV-Ch) | NP_620544.1 |
|  | Homalodisca vitripennis reovirus (HVRV) | ACO37232.1 |
|  | Rice gall dwarf virus (RGDV) | YP_001111373.1 |
|  | Rice gall dwarf virus GX (RGDV-GX) | ABF67520.1 |
| *Rotavirus* | Human rotavirus B (HuRV-B) | ACD39819.1 |
|  | Porcine rotavirus C (PoRV-C) | AAB00801.1 |
|  | Porcine rotavirus Co (PoRV-C/Co) | P26190.1 |
|  | Human Rotavirus C Bristol (HuRV-C/Bristol) | YP_392464.1 |
|  | Porcine rotavirus YM (PoRV/YM) | Q85036.1 |
|  | Porcine rotavirus Go (PoRV/Go) | P17699.1 |
|  | Rotavirus A Hu/Dhaka6 (RV-A/Hu/Dhaka6) | ABU41827.1 |
|  | Simian rotavirus A/SA11 (SiRV-A/SA11) | CAA34732.1 |
|  | Simian rotavirus A/SA11-both (SiRV-A/SA11-B) | P22678.1 |
|  | Simian rotavirus A/SA11-H96 (SiRV-A/SA11-H) | A2T3S0.1 |
|  | Italian lapine rotavirus 30/96 (ILRV-30/96) | ABC66297.1 |
|  | Lamb rotavirus (LaRV) | ACN18216.1 |
|  | Bovine rotavirus UKtc (BoRV-UKtc) | CAA39085.1 |
|  | Simian rotavirus A strain RRV (SiRV-A/RRV) | ABQ59568.1 |
| *Mimoreovirus* | Micromonas pusilla reovirus (MpRV) | YP_654545.1 |
| *Aquareovirus* | Atlantic salmon reovirus TS (ASRV-TS) | ABO32573.1 |
|  | Chum salmon reovirus CSV (CHSRV) | AAL31497.1 |
|  | SMReV | HM989931 |
|  | American grass carp reovirus (AGCRV) | ABV01040.1 |
|  | Grass carp reovirus 873 (GCRV-873) | AAG10436.1 |
|  | Golden shiner reovirus (GSRV) | AAM92745.1 |
|  | Grass carp reovirus HZ08 (GCRV-HZ08) | ADJ75336.1 |
| *Orthoreovirus* | Avian orthoreovirus (ARV) | ACH72475.1 |
|  | Mammalian orthoreovirus 2 (MRV-2) | ABG49449.1 |
|  | Mammalian orthoreovirus 3 (MRV-3) | ABP48913.1 |
|  | Mammalian orthoreovirus 1 (MRV-1) | NP_694626.2 |
| *Cypovirus* | Dendrolimus punctatus cypovirus 1 (DpCPV-1) | AAN46860.1 |
|  | Lymantria dispar cypovirus 1 (LdCPV-1) | AAK73521.1 |
|  | Bombyx mori cypovirus 1 (BmCPV-1) | AAK20302.1 |
|  | Heliothis armigera cypovirus 14 (HaCPV-14) | ABB51571.1 |
|  | Lymantria dispar cypovirus 14 (LdCPV-14) | AAK73087.1 |
| *Dinovernavirus* | Aedes pseudoscutellaris reovirus (APRV) | YP_443936.1 |
| Oryzavirus | Rice ragged stunt virus Thailand (RRSV-Tai) | NP_620541.1 |
| *Coltivirus* | Eyach virus fr578 (EYAV-Fr578) | NP_620280.1 |
|  | Colorado tick fever virus florio (CTFV-Fl) | NP_690891.1 |
| Mycoreovirus | Mycoreovirus 1 CPRV 9B21 (CPRV-9B21) | YP_001936004.1 |
|  | Mycoreovirus 3 RaRV (RaRV) | YP_392478.1 |
| *Fijivirus* | Nilaparvata lugens reovirus (NLRV) | NP_619776.1 |
|  | Nilaparvata lugens reovirus Izumo (NLRV-Iz) | BAA08542.1 |
|  | Fiji disease virus (FDV) | YP_249762.1 |
|  | Mal de Rio Cuarto virus (MRCV) | AAO73182.1 |
